# Properties of biochar derived from wood and high-nutrient biomasses with the aim of agronomic and environmental benefits

**DOI:** 10.1371/journal.pone.0176884

**Published:** 2017-05-11

**Authors:** Rimena R. Domingues, Paulo F. Trugilho, Carlos A. Silva, Isabel Cristina N. A. de Melo, Leônidas C. A. Melo, Zuy M. Magriotis, Miguel A. Sánchez-Monedero

**Affiliations:** 1 Department of Soil Science, Universidade Federal de Lavras, UFLA, Lavras, Minas Gerais, Brazil; 2 Forest Sciences Department, Universidade Federal de Lavras, UFLA, Lavras, Minas Gerais, Brazil; 3 Chemistry Department, Universidade Federal de Lavras, UFLA, Lavras, Minas Gerais, Brazil; 4 Centro de Edafología y Biología Aplicada del Segura (CEBAS-CSIC), Departmento de Conservación de Suelos y Agua y Manejo de Residuos Orgánicos, Campus Universitario de Espinardo, Murcia, Spain; RMIT University, AUSTRALIA

## Abstract

Biochar production and use are part of the modern agenda to recycle wastes, and to retain nutrients, pollutants, and heavy metals in the soil and to offset some greenhouse gas emissions. Biochars from wood (eucalyptus sawdust, pine bark), sugarcane bagasse, and substances rich in nutrients (coffee husk, chicken manure) produced at 350, 450 and 750°C were characterized to identify agronomic and environmental benefits, which may enhance soil quality. Biochars derived from wood and sugarcane have greater potential for improving C storage in tropical soils due to a higher aromatic character, high C concentration, low H/C ratio, and FTIR spectra features as compared to nutrient-rich biochars. The high ash content associated with alkaline chemical species such as KHCO_3_ and CaCO_3_, verified by XRD analysis, made chicken manure and coffee husk biochars potential liming agents for remediating acidic soils. High Ca and K contents in chicken manure and coffee husk biomass can significantly replace conventional sources of K (mostly imported in Brazil) and Ca, suggesting a high agronomic value for these biochars. High-ash biochars, such as chicken manure and coffee husk, produced at low-temperatures (350 and 450°C) exhibited high CEC values, which can be considered as a potential applicable material to increase nutrient retention in soil. Therefore, the agronomic value of the biochars in this study is predominantly regulated by the nutrient richness of the biomass, but an increase in pyrolysis temperature to 750°C can strongly decrease the adsorptive capacities of chicken manure and coffee husk biochars. A diagram of the agronomic potential and environmental benefits is presented, along with some guidelines to relate biochar properties with potential agronomic and environmental uses. Based on biochar properties, research needs are identified and directions for future trials are delineated.

## Introduction

Large amounts of crop residues are generated worldwide and they are not always properly disposed of or recycled. Wood log production in Brazil generates about 50.8 million m^3^ of lignocellulosic residue yearly [1], while nearly 200 million tons/year of sugarcane bagasse is generated [2]. In 2016, 49 million bags of coffee [3] were harvested and almost the same amount (by weight) of coffee husk was produced. Based on the Brazilian chicken flock and on the average amount of manure produced per animal, about 12 million t year^-1^ of manure were generated in Brazil in 2009 [1]. Chicken manure is characterized by high N, P, Ca, and micronutrient contents, while coffee husk contains the highest K concentration [4]. Sugarcane bagasse and wood-derived wastes have low amounts of nutrients and high lignin and cellulose content.

In humid tropical areas, the application of raw residues on soils is the main management practice, but this has limited impact on increasing C in soils due to high organic matter decomposition rates [5]. *In natura* disposal of coffee husk in crop fields may lead to an increased population of *Stomoxys calcitrans*, a pest that may cause damages to dairy cattle and feedlots [6]. Conversion of wastes into biochar increases the recalcitrance of C due to increased proportions of condensed aromatic compounds in the biochare, which ensures higher persistence of C in the soil compared to the C from raw biomass [7]. In addition, conversion of wastes into biochar reduces residue volume, generates energy, improves the efficiency of nutrient use by crops, eliminates pathogens, and generates products with high agronomic value [8–10].

Characterization of biochars generated from the main Brazilian organic wastes is the first step in identifying agronomic and environmental applications and guiding future research trials. Plant-derived biochars have high aromatic C content due to the greater amount of lignin and cellulose present, which gives the biochar high stability and resistance to microbial decomposition [11]. Animal manures have high contents of labile organic and inorganic compounds, resulting in biochars with high ash content, which is positively related to the nutrient and chemical composition of the biomass [8, 12]. Higher ash, N, S, Na, and P concentration have been observed in poultry litter biochar than in peanut hull and pecan shell biochars [13]. High nutrient concentrations in the biomass can generate biochars with more ash content and alkalizing capacity [14]. Thus, biochar can be used in soils to correct acidity [12], increase soil cation exchange capacity (CEC), retain water [15–16, 12], and regulate C and N dynamics [17]. In addition, researchers have pointed out positive effects of biochar on soil remediation due to its adsorption of pesticides or metals [18–20].

We characterized biochars derived from wood, sugarcane bagasse, and nutrient-rich residues (coffee husk, chicken manure) aiming to identify potential agronomic and environmental benefits for fertilizing soil and enhancing soil quality. Our hypothesis is that nutrient-rich biochars derived from waste have fertilization potential, while biochars derived from wood and sugarcane charred at high temperature are potential for increasing C sequestered in soils. We also hypothesized that the liming value of the biochar is primarily regulated by its ash content, regardless of its pH; the mineral phase of chicken manure is effective in protecting the organic compounds from degradation, ensuring production of high CEC biochars even under high temperature (750°C). In this study, we aimed to (i) assess the chemical and physicochemical properties of biochars derived from wood and nutrient-rich sources in terms their potential agronomic and environmental benefits, and (ii) identify potential uses and drawbacks in biochar production from contrasting biomass types and suggest guidelines for future research trials in biochar-treated soils.

## Materials and methods

### Biochar manufacture

Fifteen biochars were produced from five biomass and three pyrolysis temperatures (350, 450, and 750°C). The biomasses selected were those with greatest availability in Brazil: i) chicken manure (CM); ii) eucalyptus sawdust (ES); iii) coffee husk (CH); iv) sugarcane bagasse (SB); and v) pine bark (PB). The nutrient concentrations of the biomasses are shown in S1 Table.

The biochars were produced by a slow pyrolysis procedure in an adapted muffle furnace with a sealed chamber to prevent airflow. Prior to pyrolysis, biomass wasoven dried at 105°C. The amount of material used in each procedure varied according to the density of each material. A heating rate of 1.67°C min^-1^ was adopted, and the final temperature reached were 350, 450, and 750°C. The target temperature was maintained for 30 minutes and the biochar sample was cooled to room temperature. The yield of the biochar mass was calculated as follows:

Yield (%)=[100 x (biochar mass ÷ 105°C dried biomass)] (1)

### Biochar characterization

#### Yield and ash content

The volatile material, ash, and fixed carbon concentrations were determined according to standard procedure D-1762-84, established by the American Society for Testing and Materials [21]. The biochar samples (< 0.25 mm) were oven dried at 105°C and then heated in a covered crucible inside a muffle furnace at 950°C for 6 minutes. The resulting loss of mass refers to volatile material (VM). The biochar was then returned to the oven and heated in an open crucible at 750°C for 6 hours. The mass of material remaining after incineration refers to ash. Finally, the fixed carbon (FC) concentration was determined by the following equation:

FC (%)=[100−(VM+Ash)] (2)

Thermogravimetric analysis (TGA) was performed using a Shimadzu DTG-60H device. Samples of approximately 5 mg were heated from room temperature to 600°C at a rate of 10°C min^-1^ and a nitrogen flow of 50 mL min^-1^. Then, the first derivative of the TGA curve was calculated, which establishes loss in mass over the temperature range employed.

#### Biomass and biochar elemental composition

The elemental composition (C, H, N, S) of the biochars was determined on 0.5 g of ground and sieved (200 mesh) material by dry combustion using TOC and CHNS analyzers (Vario TOC cube, Elementar, Germany). Biochar oxygen concentrations were obtained by difference as follows:

O(%)=[100−(C+H+N+S+Ash)] (3)

The biochar elemental composition was used to calculate the H/C, O/C, and (O + N)/C ratios [22].

Water-soluble organic carbon (WSOC) and water-soluble inorganic carbon (WSIC) was measured in a 10% (w v^-1^) biochar-water mixture shaken for 1 h and then filtered through a 0.45 μm membrane filter. In the liquid extracts, WSOC and WSIC were quantified using the liquid mode of a TOC analyzer (Vario TOC cube, Elementar, Germany). Considering that a single 1 h extraction is unlikely to solubilize all water-soluble organic and inorganic C from biochar, it should be take into account that WSOC and WIOC provide an index of part of water soluble C chemical species rather than 100% of all biochar soluble C; however, they were considered suitable for comparisons among biochars.

#### ATR-FTIR analysis

Fourier transform infrared spectroscopy (FTIR) analysis was performed on a Perkin Elmer Spectrum 1000 device equipped with an attenuated total reflectance (ATR) accessory, in which the powder of each sample was inserted in a diamond crystal gate. All biomass and biochars had been dried at 65°C and sieved through a 0.150 mm mesh. FTIR spectra from 32 scans was recorded in the wavenumber range 4000–500 cm^-1^ with 2 cm^-1^ resolution. The broad band chemical group assignments described in Jindo et al. [23] were used to interpret the FTIR-ATR spectra.

#### X-ray diffraction

The X-ray diffraction (XRD) analysis was carried out at the XRD1 beam-line of the Brazilian Synchrotron Light Laboratory (LNLS), Campinas, SP, Brazil, for detection of all mineral phases present in the biochars. Powdered biochar samples (< 150 mesh) were inserted in glass capillaries and analyzed in the X-Ray diffractometer through the range of 4–60° 2ɵ in a transmission mode with steps of 0.2° 2ɵ and a wavelength of about 1.0 Å. Minerals found in the biochar structure were identified after calculation of the *d* spacing according to Bragg’s law. The peak areas identified for different minerals were compared with XRD patterns of standard minerals compiled by the Mineralogy Database available at “web minerals” (http://webmineral.com/).

#### Chemical and physicochemical attributes

Biochar pH was measured in deionized water and in a 0.01 mol L^-1^ CaCl_2_ solution at a 1:10 (w/v) ratio, after shaking the samples for 1h. All measurements were performed in triplicate. Biochar CEC was determined by the modified ammonium acetate compulsory displacement method, adapted to biochars [24]. During CEC determination, a vacuum filtration system was employed, and samples were filtered through a 0.45 um membrane filter. Initially, 0.5 g of biochar sample was leached five times with 20 mL of deionized water to remove excess salts. After that, the samples were washed three times with a 1 mol L^-1^ sodium acetate (pH 8.2) solution, followed by five washes with 20 mL of ethanol to remove free (non-sorbed) Na^+^ ions. Samples were then washed four times with 20 mL of 1 mol L^-1^ ammonium acetate to displace the Na^+^ from the exchangeable sites of the biochar. The leachates were collected and stored in a 100 mL volumetric flask, and Na contents in the leachates were determined by flame photometry. The CEC corresponds to the amount of Na adsorbed per unit mass of biochar, expressed as cmol_c_ kg^-1^.

The biochar liming value (LV) was evaluated by the acid-base titration method [25]. A quantity of 0.5 g of biochar (< 0.25-mm) was placed in a 50 mL plastic bottle, and then 20 mL of distilled water was added. The bottles were stirred for 2 h and then titrated with 0.1 mol L^-1^ of HCl solution to a pH 2.0 end point. To ensure that the biochar pH was stabilized at 2.0, after 12 h of equilibration, the pH was again measured and, if necessary, corrected with the HCl solution already mentioned. Based on the assumption that alkalinity is the capacity of biochar to accept protons from a 0.05 M HCl solution (1.3 ≤ pH ≤ 2) after 72 h of equilibration [26], LV is a partial measurement of biochar total alkalinity. The volume of acid used and its pH value were recorded. These results were used to calculate the LV, here defined as the volume of 0.1 mol L^-1^ HCl necessary to reduce the biochar pH by one unit, according to the following equation:

Liming value (volume of HCl/pH unit) = (total volume of HCl to reach the titration end point/pH interval).

### Experimental design and statistical analysis

Biochars are hereby referred by the biomass abbreviation and pyrolysis temperature, for example, CH350 denotes coffee husk pyrolysed at 350°C and CH750, coffee husk pyrolysed at 750°C. The experimental design used was factorial completely randomized with five biomasses (CM, ES, CH, SB, PB) combined with three pyrolysis temperature (350, 450, 750°C).

The data were subjected to analysis of variance (ANOVA) for significant differences between factors as biomasses, pyrolysis temperatures, and their interaction. When significant F-tests were obtained (0.05 probability level), the factors separation was achieved using Tukey’s honestly significant difference test. Data were statistically analysed employing SISVAR [27].

## Results and discussion

### Yield, volatile matter, and ash content

Biochar yields were reduced and ash contents increased with an increase in pyrolysis temperature (Table 1). The CM biochar at three temperatures (350, 450 and 750°C) showed higher yield and higher ash content than the other biochars (Table 1), due to large amount of inorganic compounds (K, P, Ca, and Mg) in this biomass (S1 Table), which accumulated after volatilization of C, O, and H compounds. Coffee husk biochar also showed a high ash content, which is probably due to the high K (22 g kg^-1^) content of the biomass. The ES and SB biochars, regardless of the pyrolysis temperature, showed the lowest ash content (<1.1% and <2.2%, respectively) (Table 1), explained by their low nutrient content (S1 Table). According to derivative thermogravimetric (DTG) curves of biomass losses (S1 Fig), ES and SB showed higher mass loss between 250 and 350°C, which is attributed to high cellulose content in the biomass [28], which is easily degraded during low-temperature pyrolysis. CM, CH and, PB biochars showed lower mass loss between 250 and 350°C indicating higher thermal stability (S1 Fig).

**Table 1:** Yield and proximate analysis (volatile matter, ash, carbon fixed) of biochars produced at different pyrolysis temperatures.

Biomass	Temp. (°C)	Yield (%)	Proximate analysis (wt. %)		
			Volatile Matter	Ash	Carbon Fixed
Chicken manure	350	69.7	36.9 Ab	52.0 Ba	11.1 Cd
	450	63.0	30.6 Ba	55.3 Aa	14.1 Be
	750	55.9	26.5 Ca	56.4 Aa	17.0 Ae
Eucalyptus sawdust	350	42.5	36.9 Ab	0.9 ABe	62.2 Cb
	450	36.0	28.5 Bb	0.7 Be	70.8 Bb
	750	28.2	6.5 Cd	1.1 Ae	92.4 Aa
Coffee husk	350	43.5	34.6 Ac	12.9 Bb	52.5 Cc
	450	37.7	26.2 Bc	12.9 Bb	60.9 Bc
	750	31.6	17.6 Cb	19.6 Ab	62.8 Ad
Sugarcane bagasse	350	37.5	35.0 Ac	1.9 Ad	63.0 Ca
	450	33.2	24.0 Bd	2.1 Ad	73.9 Ba
	750	26.9	7.7 Cc	2.2 Ad	90.1 Ab
Pine bark	350	59.6	38.5 Aa	8.3 Bc	53.2 Cc
	450	49.3	29.3 Ba	7.9 Bc	62.8 Bc
	750	38.9	6.0 Cd	14.5 Ac	79.4 Aa

Uppercase letters compare pyrolysis temperatures within the same biomass and lowercase letters compare biomass at the same temperature. The same letter do not differ by the Tukey test at *p* <0.05.

Biochar volatile matter values reduced as the pyrolysis temperature was raised from 450°C to 750°C (Table 1). This is explained by an the increase in aromatization and greater losses of gas products, tar oil and low molecular weight hydrocarbons as a result of increasing pyrolysis temperature [28]. CM750 and CH750, however, showed the smallest losses of volatiles (Table 1) in contrast to the other biochars prepared at this same temperature. This was coincident with higher quantities of ash found in these biomasses, which can protect the organic fraction and structures of biochars during pyrolysis [29–31]. Chemical activation of KOH impregnation has a catalytic effect in intensifying hydrolysis reactions, increasing volatile products [32, 33] and the development of pores in the charcoal structure [31], suggesting a role for pores in the adsorption of volatile materials [33]. Fixed C was inversely correlated with the ash contents and was higher in eucalyptus sawdust and sugarcane bagasse biochar compared to other biochars produced (Table 1).

### Elemental composition and soluble C fractions

Total C concentrations in plant-derived biochars increased with an increase in pyrolysis temperature (Table 2), whereas the O and H concentrations diminished (Table 2). Biochars derived from plant biomass showed the highest C concentration, up to 90% C for ES and SB pyrolyzed at 750°C (Table 2). Increase in C concentrations with a rise in pyrolysis temperature occurs due to a higher degree of polymerization, leading to a more condensed carbon structure in the biochar [11]. Similar results were reported for biochars produced from pine straw [22], peanut shells [13], sugarcane bagasse [34], and wheat straw [35]. The greater the degree of formation of aromatic structures is, the higher the resistance of the biochar to microbial degradation [36, 7]. The C concentration in CM biochar reduced with an increase in pyrolysis temperature (Table 2). Such results suggest that the organic compounds found in animal waste are more labile and are rapidly lost as pyrolysis temperature is increased, before the formation of biochar with recalcitrant compounds. A 6% reduction in C concentration in poultry litter biochar was reported when pyrolysis temperature was increased from 350°C to 700°C [8], as well as a decrease in sewage sludge biochar C content [37]. The C concentration in CM biochar was lower (≈ 30% C) than wood biochars (Table 2). These results are in agreement with those of Novak et al. [13].

**Table 2:** Elemental composition (C, H, S, O), and atomic ratios (H/C, O/C) of biochars produced at different pyrolysis temperatures.

Biomass	Temp. (°C)	Elemental composition (%)				Atomic ratio	
		C	H	S	O	H/C	O/C
Chicken manure	350	31.2 Ad	1.97 Ac	0.31 Ba	10.9 Bc	0.76 Ba	0.26 Ba
	450	27.2 ABd	1.92 Bc	0.44 Aa	11.4 Bc	0.85 Aa	0.31 Ba
	750	24.7 Bd	0.67 Cc	0.29 Ba	16.3 Aa	0.32 Ca	0.49 Aa
Eucalyptus sawdust	350	70.4 Ca	3.81 Ab	0.02 Ac	24.0 Aab	0.65 Aa	0.26 Ab
	450	78.6 Ba	3.42 Ba	0.01 Ac	16.6 Bab	0.52 Bb	0.16 Bc
	750	90.9 Aa	1.52 Ca	0.04 Ac	5.6 Cc	0.20 Cc	0.05 Cc
Coffee husk	350	60.5 Bc	3.92 Ab	0.09 Bb	19.5 Aab	0.78 Aa	0.24 Aa
	450	61.3 Bc	3.65 Ba	0.10 Bb	19.0 Aa	0.71 Aa	0.23 Ab
	750	66.0 Ac	1.57 Ca	0.23 Ab	9.8 Bb	0.29 Bb	0.11 Bb
Sugarcane bagasse	350	74.7 Ca	4.26 Aa	0.03 Ac	17.9 Ab	0.68 Aa	0.18 Ab
	450	81.6 Ba	3.66 Ba	0.05 Ac	11.3 Bbc	0.54 Bb	0.10 Bc
	750	90.5 Aa	1.64 Ca	0.06 Ac	4.3 Cc	0.22 Cc	0.04 Cc
Pine bark	350	67.6 Cb	3.73 Ab	0.01 Ac	28.7 Aa	0.66 Aa	0.32 Aa
	450	75.2 Ba	2.74 Bb	0.02 Ac	24.7 Ba	0.44 Bb	0.25 Bc
	750	86.3 Aab	1.16 Cb	0.04 Ac	19.1 Ca	0.16 Cc	0.17 Cc

Uppercase letters compare pyrolysis temperatures within the same biomass and lowercase letters compare biomass at the same temperature. The same letter do not differ by the Tukey test at *p* <0.05.

The H/C and O/C ratios of biochars derived from plant biomass decreased as the pyrolysis temperature was increased (Table 2), indicating increasing aromaticity and a lower hydrophilic tendency, respectively [8, 13]. An increase in the aromatic character of biochars is associated with dehydration reactions and removal of O and H functional groups, as well as the formation of aromatic structures, as charring is intensified [11]. These features are consistent with the van Krevelen diagrams generated in this study, which showed a positive relationship between H/C and the O/C atomic ratios (S2 Fig). Biochars derived from CM did not change H/C and O/C ratios or the degree of aromaticity as the pyrolysis temperature increased from 350 to 450°C (Table 2).

**Fig 1:**
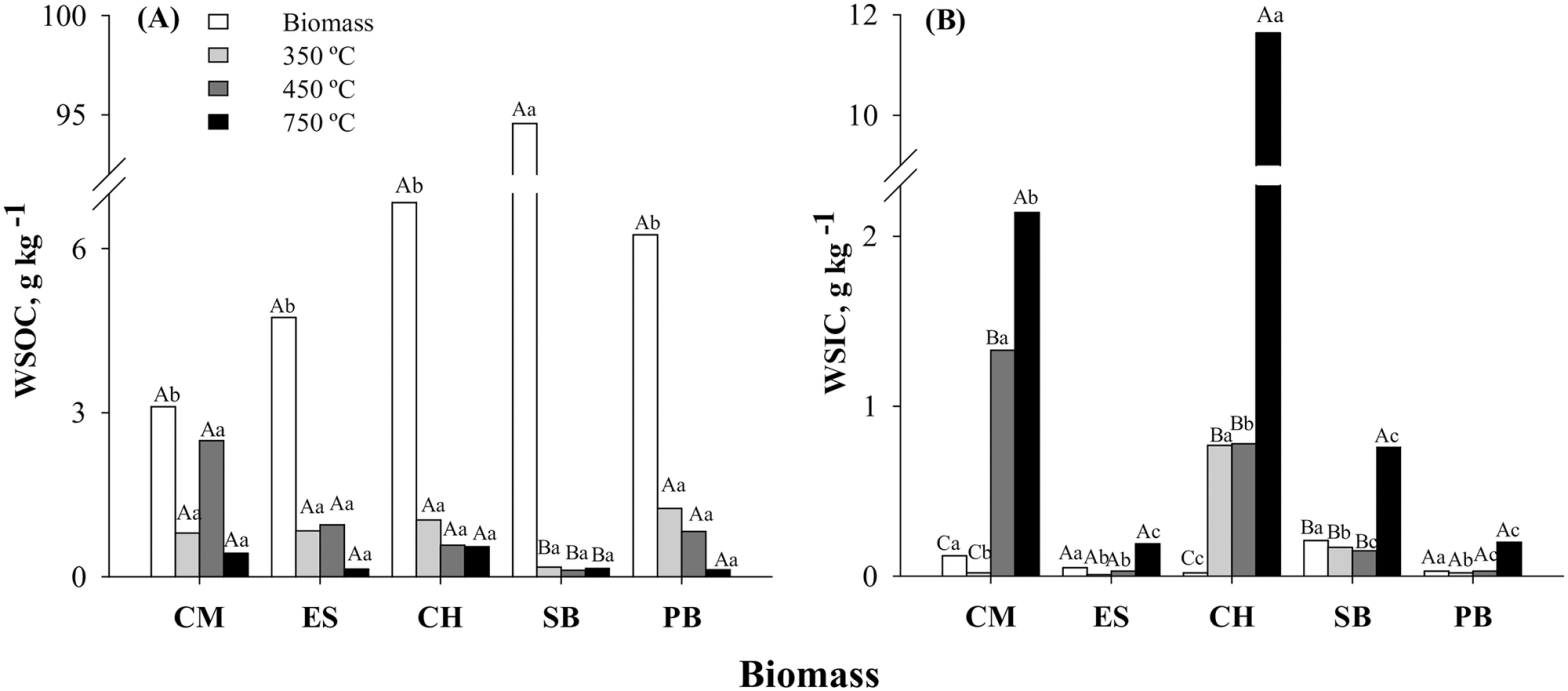
Water-soluble organic carbon—WSOC (A) and water-soluble inorganic carbon—WSIC (B) of biomasses and biochars at different pyrolysis temperatures. CM = chicken manure, ES = eucalyptus sawdust, CH = coffee husk, SB = sugarcane bagasse, and PB = pine bark. Uppercase letters compare pyrolysis temperatures within the same biomass and lowercase letters compare biomass at the same temperature. Bar followed by the same letter do not differ by the Tukey test at *p* <0.05.

The sugarcane bagasse biomass had the highest WSOC concentration (94.5g kg^-1^) (Fig 1A). However, with increasing pyrolysis temperature, WSOC concentration in bagasse were significantly reduced (< 0.2g kg^-1^), suggesting that the water-soluble carbon is degraded or incorporated into the organic compounds of biochar even at a relatively low pyrolysis temperature.

**Fig 2:**
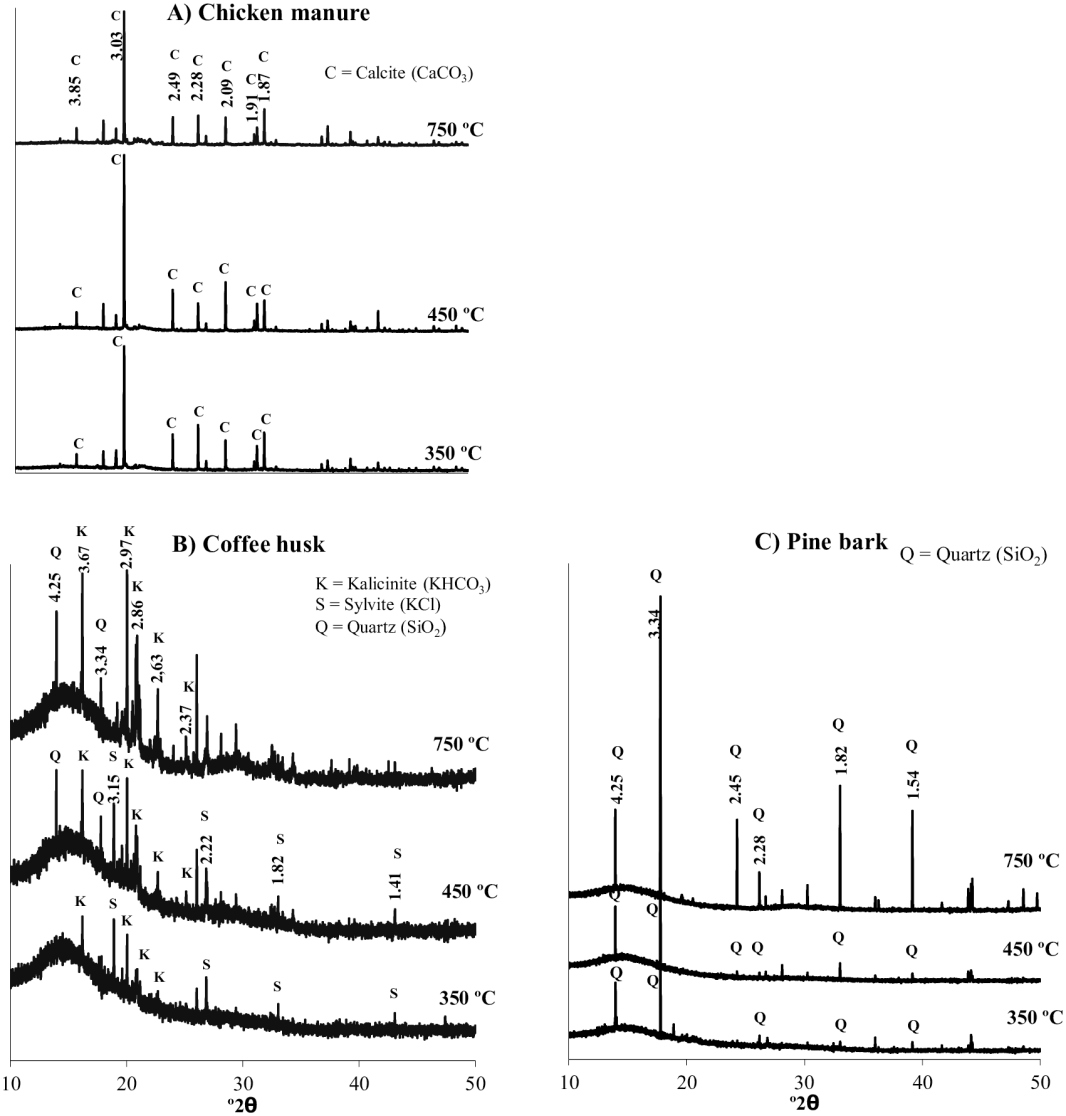
X-ray diffraction spectra of biochars pyrolized at different temperatures (350, 450 and 750°C). (A) Chicken manure biochar. (B) Coffee husk biochar. (C) Pine bark biochar.

The biochar WSIC concentration increased with pyrolysis temperature (Fig 1A). The highest WSIC concentration (11.7g kg^-1^) was verified for CH750. WSIC-coffee biochar was significantly (p<0.05) different from the other biochars produced at other pyrolysis temperatures. The WSIC concentrations of CM and SB biochars were also influenced by the pyrolysis temperature, especially those samples pyrolyzed at 750°C, whose WSIC concentration were 2.1 g kg^-1^ and 0.8 g kg^-1^, respectively (Fig 1B). For the other biochar samples, the WSIC concentration was not significantly (p<0.05) different (Fig 1B). The higher WSIC concentration found in CH750 in comparison with similar low-temperature biochar is probably due to the presence of the mineral kalicinite (Fig 2), a K inorganic compound with high solubility in water [38].

### Spectroscopic characterization

#### X-ray diffractometry

Mineral components in the crystal form were identified in the CM, CH and PB biochars (Fig 2). No crystal substances were observed in the X-ray diffraction spectra for ES and SB biochars. For CM biochars produced at all temperatures, the presence of calcite (CaCO_3_) was identified by peaks at 3.85, 3.03, 2.49, 2.28, 2.09, 1.91, and 1.87 Å (Fig 2A). The presence of calcite in CM biochars is consistent with the high Ca content found in the chicken manure biomass (S1 Table). The presence of calcite in this biochar sample is probably due to the addition of phosphogypsum in manure, normally used to stabilize N forms during composting [4], as well as the use of calcium carbonate in chicken diets. Similarly, calcite and dolomite [CaMg(CO_3_)_2_] were identified in sewage sludge biochar at 300–800°C [39].

For all CH biochars, the presence of kalicinite (KHCO_3_) was observed (Fig 2B). The formation of KHCO_3_ may have been favored by the reaction of K with CO_2_ released during thermal decomposition of hemicellulose and cellulose [32]. An increase in the amounts of KHCO_3_ may also explain the high WSIC contents found in CH biochars (Fig 1). The peak intensity at 3.67 Ǻ increased with increasing pyrolysis temperature, indicating relative accumulation of kalicinite in CH biochars. The peaks at 3.15, 2.22, 1.82, and 1.41 Ǻ were found in CH350 and CH450 were attributed to the presence of sylvite (KCl) (Fig 2B). In durian shell biochar, kalicinite was also the dominant mineral [38]. The presence of quartz (SiO_2_) was also confirmed in CH450 and CH750 from peaks at 3.34 and 4.25 Ǻ in the X-ray spectra. Identification of SiO_2_ was also noted in the biochars produced from PB biochar at the three pyrolysis temperatures (Fig 2B). Yuan et al. [25] also identified the presence of sylvite and calcite in biochars from canola straw pyrolyzed at 300, 500, and 700°C.

#### FTIR analysis

**Fig 3:**
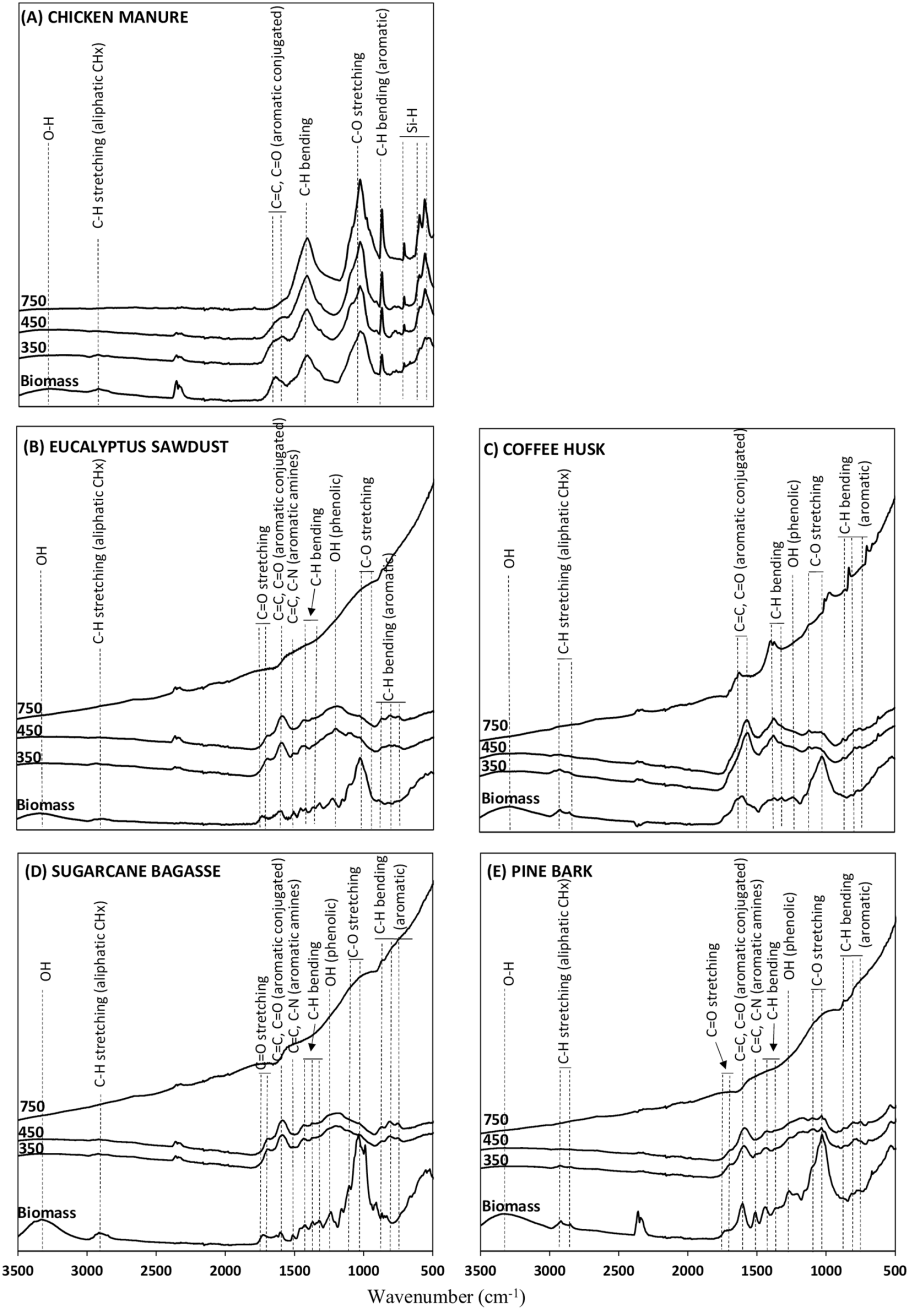
FTIR-ATR spectra of biomasses and their respective biochars pyrolyzed at 350, 450, and 750°C. (A) Chicken manure. (B) Eucalyptus sawdust. (C) Coffee husk. (D) Sugarcane bagasse. (E) Pine bark.

The FTIR-ATR biomass and biochar spectra are shown in Fig 3. The spectra of the all biomass samples showed a broad band at 3200–3400 cm^-1^, which is attributed to -OH from H_2_O or phenolic groups [22, 40, 11]. For all biomass sources, absorption in the region between 2920 and 2885 cm^-1^ (C-H stretching) was assigned to aliphatic functional groups [8, 40, 11], and the strong band at 1030 cm^- 1^ is due to the C-O stretching and associated with oxygenated functional groups of cellulose, hemicellulose, and methoxyl groups of lignin [8, 35, 41] [3–5]. The intense bands at 1270 cm^−1^ were assigned to phenolic—OH groups [22].

Changes in biochar organic structure were apparent when biomass was pyrolyzed at 350°C, except for the CM biochars (Fig 3). The intensities of bands of -OH (3200–3400 cm^-1^), aliphatic C-H stretching (2920 and 2885 cm^-1^), -OH phenolic (1270 cm^-1^), and C-O stretching region (1030 cm^-1^) decreased sharply due to degradation and dehydration of cellulosic and ligneous components, even at low temperatures (350°C) [35, 22]. An increase in band intensity in the 1600 cm^-1^ region (C = C, C = O of conjugated ketones and quinones) and the appearance of weak bands between 885 and 750 cm^-1^ (aromatic CH out-of-plane) were attributed to an increasing degree of condensation of the biochar organic compounds. An increase in the degree of biochar condensation as pyrolysis temperature increases is in agreement with the results reported by Keiluweit et al. [35], Jindo et al. [23], and Melo et al. [40]. In the FTIR spectra of ES750, SB750, and PB750 biochars most of the organic functional groups present in the biochar structure were lost (Fig 3B, 3D and 3E). For CH biochars, weak bands remaining at the highest pyrolysis temperature were identified, which were assigned to aromatic C = C stretching (at about 1600 cm^-1^), -C-H_2_ bending (1400 cm^-1^), and aromatic C-H bending (885 cm^-1^). Losses of chemical groups in CH750 could explain the sharp decrease in CEC of this biochar in comparison to CH350 and CH450. In the CM biochars, the intensity of all organic functional bands remained largely unchanged after the biomasses were subjected to the charring process, regardless of the pyrolysis temperature used (Fig 3A). Protection of organic groups, even at high pyrolysis temperature, may be associated with the high ash content found in coffee husk and chicken manure (Fig 3). Ash acts as a heat resistant component, which may protect organic compounds against degradation and may hinder the formation of aromatic structures as charring intensity advances [42].

### Physicochemical properties

**Fig 4:**
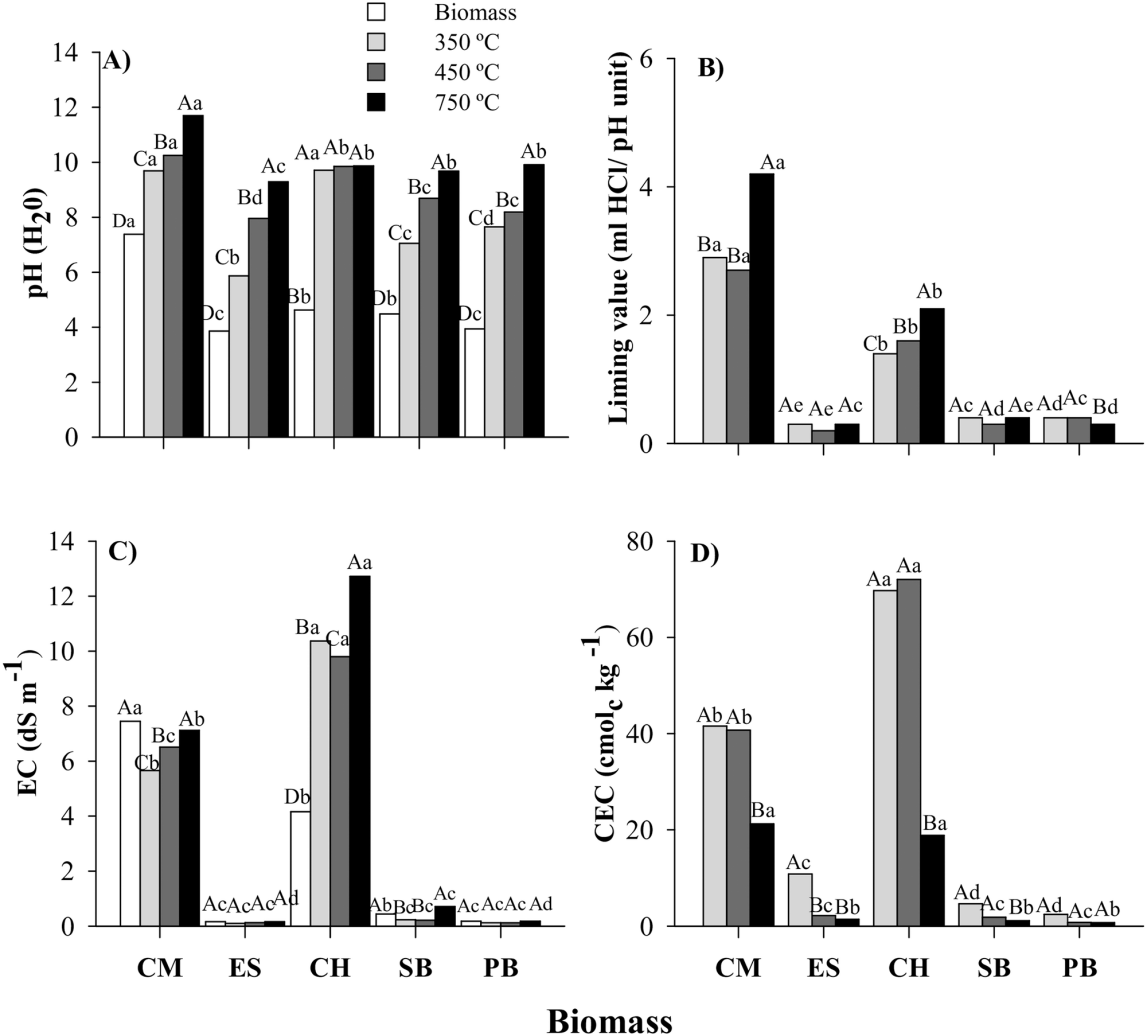
Values of pH-H_2_0 (A), liming value (B), EC—electrical conductivity (C), and CEC—cation exchange capacity (D) as related to biomass and biochars. CM = chicken manure, ES = eucalyptus sawdust, CH = coffee husk, SB = sugarcane bagasse, and PB = pine bark. Uppercase letters compare pyrolysis temperatures within the same biomass and lowercase letters compare biomass at the same temperature. Bar followed by the same letter do not differ by the Tukey test at *p* <0.05.

The pH in water of the biochars ranged from slightly acidic to alkaline (Fig 4A). Overall, the pH values of biochars were higher than 6.0 units. Compared to the biomass pH, the charring process increased pH in water and, in some cases, differences were up to 4 pH units for some of the biomasses pyrolyzed at 750°C (Fig 4A). An increase in biochar pH with pyrolysis temperature has been reported for corn straw [25], sewage sludge [36], pine [43], poultry litter [44], and sugarcane straw [40] biochars. With increasing temperature, there is an enrichment of basic cations in the ashes, which may be associated with alkaline species, such as carbonates, oxides and hydroxides [25, 45], and a reduction in the concentration of acidic surface functional groups [16]. Among biochars, the highest pH values were recorded for the CM biochars, which exhibited a pH of 9.7 (at 350°C), 10.2 (at 450°C), and 11.7 (at 750°C) (Fig 4A). In general, all biochars pyrolyzed at 750°C showed pH values higher than 8.0.

Biochars of ES, SB, and PB produced at all pyrolysis temperatures used in this study showed reduced liming values (capacity to neutralize acidity) (Fig 4B), i.e, the ability to correct soil acidity should not only be evaluated by the pH value. CM and CH biochars, regardless of the pyrolysis temperature, showed higher liming values compared to the other biochars (4B), which were related to the high mineral concentration in chicken manure and coffee biochars, specifically to the calcium and potassium carbonates found in their respective X-ray diffraction spectra (Fig 2A and 2B). The presence of carbonates has been previously reported as the main alkaline components of the biochars [25]. Biochars produced from tomato [46] and paper sludge [16] showed high liming value, which was attributed to the presence of calcite and other carbonate minerals in these biochars. Thus, the biochar liming value is mainly regulated by the biochar ash content and chemical composition (especially of basic cations) and, to a much lesser extent, by the biochar pH. This characteristic should be considered when biochar is added to soils to correct soil acidity.

Electrical conductivity (EC) was mainly influenced by the biomass used in biochar production (Fig 4C). At all pyrolysis temperatures, the CH biochar showed the highest EC value, followed by the CM biochar (Fig 4C). These results, among other factors, may be due to the presence of soluble minerals, i.e., kalicinite and sylvite, in CH biochar (Fig 2B) and calcite in CM biochar (Fig 2A), and may be related to the high levels of WSIC in both biochars, as well (Fig 1B).

Biochar cation exchange capacity (CEC) values varied greatly, and are mainly dependent on the biomasses and the temperature used in the pyrolysis process (Fig 4D). CH350 and CH450 stood out from the other biochars due to the high CEC values (means of 69.7 cmol_c_ kg^-1^ at 350°C and 72.0 cmol_c_ kg^-1^ at 450°C) (Fig 4D). CM biochars produced at low temperatures (350°C and 450°C) also showed high CEC values (21.3 cmol_c_ kg^-1^) (Fig 4D). Negative charge density on biochar surfaces produced at low temperatures is attributed to the exposure of functional groups, such as carboxylic acids, ketones, and aldehydes released by depolymerization of cellulose and lignin [47, 22, 35]. CH and CM biomasses also exhibited high K concentration, which can intercalate and cause the separation of carbon lamellae by the oxidation of cross-linking carbon atoms, resulting in formation of surface groups at the edge of the carbon lamellae [32]. ES, SB, and PB biochars shown low CEC, with mean values for biochar pyrolyzed at 350°C of 10.8, 4.6, and 2.4 cmol_c_ kg^-1^, respectively (Fig 4D). An increase in pyrolysis temperature from 450°C to 750°C reduced the biochar CEC values, except for PB biochar (Fig 4D). These results were supported by the FTIR spectra shown in Fig 3, in which most of the organic group assignments and bands responsible for generating negative charges were lost, indicating the removal of oxygen-containing functional groups at most of the biochar at high temperature (750°C). Song and Guo [44] also verified that as carboxylic and phenolic group assignments disappear, the biochar CEC is lower; consequently, depending on the biomass charred, CEC is inversely correlated with pyrolysis temperature. In conclusion, biochar CEC is mainly regulated by the biomass rather than by pyrolysis temperature; however, the increase in temperature from 450°C to 750°C leads to a drastic reduction in the CEC of some biochars.

### Biochar properties related to potential environmental benefits

Carbon concentration, atomic ratios, and biochar FTIR fingerprints can be used as predictors of C persistence in biochars in soils. High C content, low H/C ratio, and FTIR spectrum features recorded for biochars derived from high temperatures are key indices of the aromatic character, stability against degradation in soils, and, consequently, high C residence time in biochar-treated soils [34, 6, 48]. Considering these, it is expected greater aromatic character for ES750, SB750, and PB750 than nutrient-rich biochars (S1 Table). As pointed out by Bruun et al. [34], the use of these biochars with a possible high residence time may be an important strategy to increase C sequestration in Brazilian soils, acting to offset greenhouse gas emissions.

In Brazil, agriculture is the main source of greenhouse gas (GHG) emissions. Most of the N_2_O emissions originate from rice fields fertilized with N and from manure deposition by cattle grazing in low and intensively managed animal production systems. Feedstock type, production temperature and process, soil properties, biochar rate, and biochar N-source interactions are the dominant factors that contribute to reductions in N_2_O emissions from biochar-treated soils [49]. In fact, Cayuela et al. [49] reported that biochar can still effective at mitigating N_2_O emissions even at pyrolysis temperatures of 400–600°C (in addition to >600°C), in application rates of 1–5%, and in coarse-textured soils with water filled pore space of <80%. In addition to the already mentioned factors, the H:Corg ratio is a suitable factor to infer the capacity of biochar in reducing N_2_O emissions [50]. According to Cayuela et al. [50], biochar with H:Corg ratio <0.3 (i.e., biochar with high degree of polymerization and aromaticity) decreased N2O emissions by 73% while biochars with H:Corg ratio >0.5 only diminished N_2_O emissions by 40%. Considering only the technical aspects, most of the 750°C biochars, and especially the wood biochars produced in this study, are potential inputs for decreasing N_2_O emissions in crop fields, but, due to the high application rates required, biochar use to offset N_2_O emissions should be focused on more profitable processes (e.g., composting) instead of use in soil.

For the purpose of reducing CO_2_ emissions, the use of low labile C biomass pyrolyzed at >550°C is recommended [50, 51]. Based on these assumptions, sugarcane bagasse, pine bark, and eucalyptus biochars pyrolyzed at 750°C are suitable for reducing CO_2_ emissions. Nevertheless, it has been suggested that the application of biochar can increase CH_4_ emissions [52, 53]. However, these studies were carried out in paddy soil, where species of methanogenic bacteria predominate and, thus, the addition of some biochars to the substrate creates a favorable environment for methanogenic microbial activity [52]. Therefore, it is very difficult to anticipate the role that may be played by the biochars characterized in this study in decreasing CH_4_ fluxes from soil to air, but wood and high-surface area biochars are potential inputs for use in soil to reduce CH_4_ emissions.

The labile C fraction in biochars can be easily decomposed and, in some cases, can stimulate the mineralization of native soil organic matter, through a positive priming effect [54, 33, 55, 50]. In general, these events occurred in soils treated with biochar produced at low temperature, but this condition may not be generalized. An increase in the biochar mineralization rate can be explained by the volatile material contained in the biochar, which may also be present in high concentrations in biochars produced at high temperatures [55]. Under these assumptions, chicken manure and coffee husk biochars both pyrolyzed at 750°C are not expected to increase C storage in soils due to their possible rapid decomposition in treated soils. The magnitude of volatile matter content in biochar is an important attribute to evaluate in C bioavailability and N cycling in biochar in the soil ecosystem. High aliphatic character (high O/C ratios and more intense FTIR peak) observed at low temperature (350 and 450°C) can be considered an index of biochar susceptibility to degradation by soil microorganisms, causing short-term immobilization of inorganic N in soil [33, 14]. This N immobilization may hamper the supply of N to plants in biochar-treated soils [56, 14]. Nevertheless, N immobilization can be seen as a beneficial mechanism for mitigating N_2_O emissions and for reducing inorganic-N leaching from soils [57, 16].

### Biochar properties related to potential agronomic benefits

**Fig 5:**
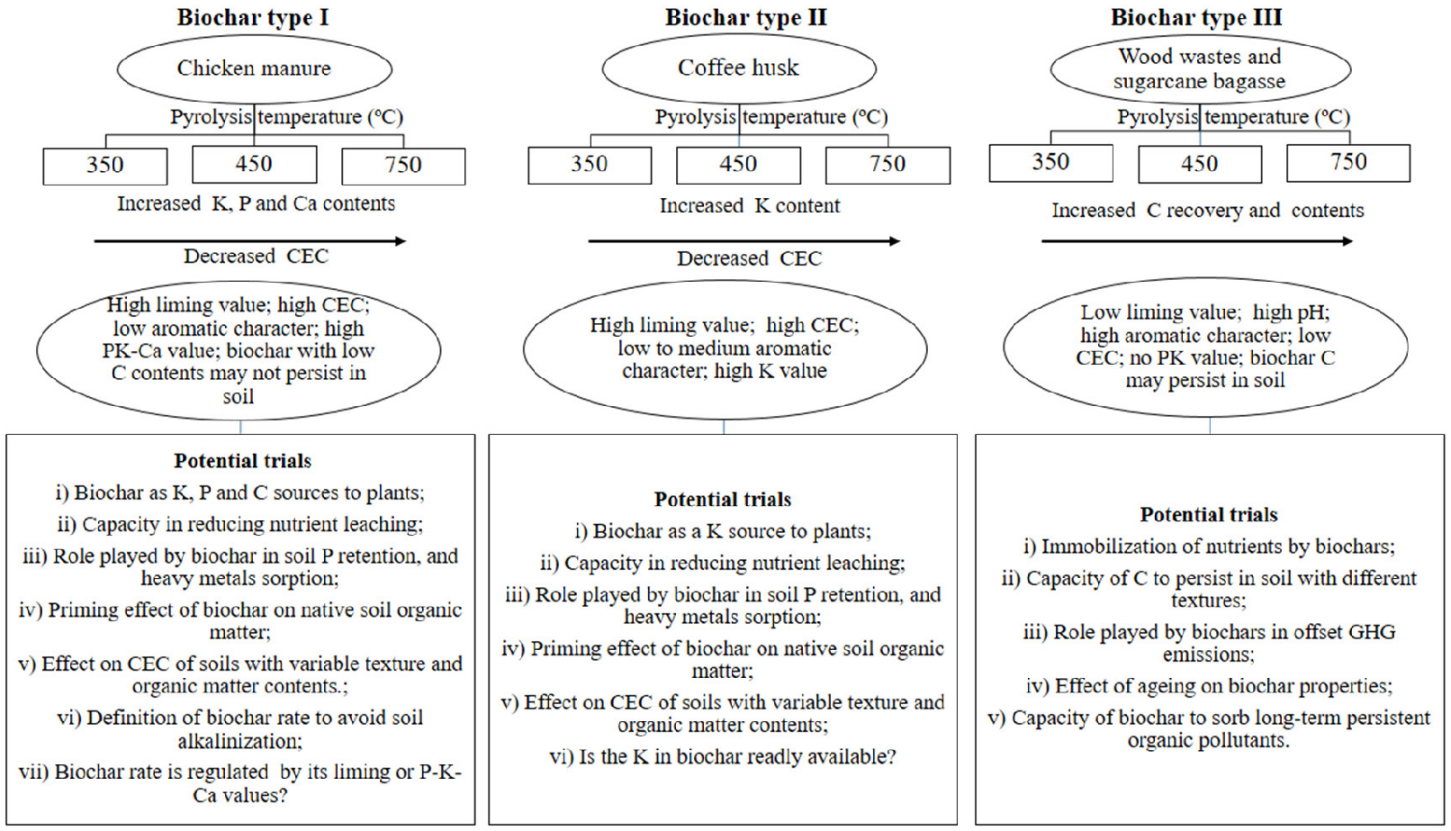
Simplified schematic representation in which wood, sugarcane, coffee husk, and chicken manure biochars are typified according to chemical and physiochemical properties and potential for carrying out trials on weathered soils in regard to their potential agronomic or environmental services.

Differentiation of biochars was established by the parameters evaluated, which allowed the identification and discussion of agronomic benefits. Characterization by proximate analysis (Table 1) showed clear differentiation in ash contents among the biochar samples. In many cases, high ash content ensures biochars rich in nutrients with high alkalizing capacity [14, 58]. The high ash content was associated with alkaline chemical species, such as KHCO_3_ and CaCO_3_, as verified by XRD analysis (Fig 2). Such characteristics make chicken manure and coffee husk biochars potential materials to increase soil acidity buffering capacity and to neutralize soil acidity, which may partially replace the large amounts of limestone used to correct soil acidity in crop fields in Brazil (Fig 5). The solubilization of these alkaline chemical species can increase soil pH, decrease Al^3+^ toxicity, reduce Fe and Mn availability, and increase soil CEC [59, 25, 60], which may decrease the precipitation and adsorption of P [61, 62], as well as enhance the supply of Ca and K to plants. The high Ca and K contents in chicken manure and coffee husk biomass (S1 Table) can significantly replace conventional sources of K (mostly imported in Brazil) and Ca, which suggests the high agronomic value of these biochars (Fig 5). However, despite the high total concentration of these chemical elements, the availability of nutrient forms in biochars should not be neglected, since an increase in pyrolysis temperature can drastically reduce the labile P forms in biochars according to [39]. Other uses of these biochars could be for remediation of some cationic trace element found in contaminated soils due to their alkalinity and high CEC (Fig 5) [63, 45, 49].

Low—temperature biochars provided the largest CEC (chicken manure and coffee husk pyrolyzed at 350–450°C), which can make them possible to adsorb N-NH_4_^+^ up to 2.3 mg g^-1^ and to reduce N leaching rates [64]. Although high-surface-area biochars generated at high temperature (>600°C) usually generate low CEC biochars, the aging effect may come into play, oxidizing the organic biochar, increasing the negative charge density and increasing the formation of biochar-mineral complexes [33].

### Recommendations and suggestions for future trials

Wood- and sugarcane-derived biochars, regardless of the charring conditions, can potentially improve C storage in tropical soils (Fig 5). The agronomic value of biochars from wastes poor in nutrients is questionable since they have low CEC, and low ash contents. Charring intensity improved the potential capacity of wood and sugarcane biochars to offset GHG emissions due to their C-fixing and aromatic character. The potential of these aromatic biochars for increasing C sequestration is probably mediated by soil texture and organic matter contents. It is more plausible to use low nutrient and high C content biochars to decrease emissions of CO_2_ rather than N_2_O, due to the high biochar rates required to offset N gas emissions from soil. The potential of biochars from wood and sugarcane bagasse for remediating contaminated soils and/or increasing water retention capacity should not be overlooked. In this case, supplementary fertilization, especially with N, should be used to avoid immobilization and maintain soil fertility [65]. In Brazil, the cost associated with the use of biochars to sequester C in soils may be offset by governmental incentives such as that offered by the Brazilian government through the Low-Carbon Agriculture (Agricultura de Baixa Emissão de Carbono—ABC) Program.

The agronomic value of the biochars generated in this study is predominantly regulated by the nutrient richness of the biomass. CM and CH biochars have high agronomic value and they should be tested in crop fields in order to identify their potential for supplying K (CH and CM) and Ca (CM) to plants and for correcting soil acidity. Several experiments have been performed trying to enrich biochars with clays and minerals to modify the final characteristics of the biochars [29, 66]. With the use of chicken manure or other nutrient-rich biomasses like coffee husk, it may be possible to create biochars to reach similar results in a natural way. Among the potential uses of biochars discussed in this study, the K content in coffee husk biochars enables them to act as a slow-release K fertilizer. Considering the average coffee husk biochar yield of 63% and a mean K_2_O content of 16% in the final coffee husk biochars, each ton of the potential organo-mineral K biochar fertilizer produced may be sold for < US$100 per ton, considering the current cost of K_2_O in Brazil (US$ 0.625/kg). In short, all the aspects and possible functions of biochars in soil emphasize the fact that the “one biochar fits all approach” [65] is not an option for the main organic wastes available in Brazil and for the biochars produced in the charring conditions of this study. Following Yargicoglu et al. [67], whatever the potential agronomic or environmental use, screening of biochars is highly recommended, given the range of variability that biomass and the extent of thermal degradation may cause in the chemical and physicochemical properties of the chars produced.

## Conclusions

In this study, the biomass source, rather than pyrolysis temperature, is the primary factor conditioning the biochar characteristics and the agronomic and environmental value of the biochar. However, pyrolysis temperature acts as a modify, changing the chemical nature and increasing the aromatic character of the organic compounds of most of the biochars investigated. In this study, characterization of the biochars was used to identify the main differences and similarities between them, offering guidelines for selecting a biomass and charring conditions to biochar end-users according to their specific soil and environmental requeriments. Biochars manufactured from ES, PB, and SB, regardless of the pyrolysis temperature employed, have potential for increasing C storage in soils, as the biochar aromatic character increases along with pyrolysis temperature. Both CH and CM biochars were also characterized by their high liming value, which make them potential materials for correcting soil acidity in crop fields. Both CH and CM biochars have a role as P and K sources for plants. High-ash biochars, such as CM and CH, produced at low-temperatures (350 and 450°C) exhibited high CEC values, which can be considered as a potential applicable material to retain nutrients. Inorganic components found in CM biochar can protect its organic compounds from degradation or hinder the charring process at 750°C. A diagram with the potential agronomic and environmental benefits of biochars is presented, and some guidelines are shown to relate the properties of biochars with their possible use. Research needs are identified and suggestions for future trials are also made.

## Supporting information

S1 Table Total nutrient contents in the biomasses investigated. ^1^The contents of P, K, Ca, Mg, Cu, Fe, Mn, and Zn were determined in extracts from the nitric-perchloric digestion procedure. ^2^Total content of B extracted with hot water.
(DOCX)

S1 Fig dTG curves of biomass. CM = chicken manure, SE = eucalyptus sawdust, CH = coffee husk, SB = sugarcane bagasse, and PB = pine bark.
(TIF)

S2 Fig van Krevelen diagram. SE = eucalyptus sawdust, CH = coffee husk, SB = sugarcane bagasse, and PB = pine bark.
(TIF)
