# Supplementary material for: Properties of biochar derived from wood and high-nutrient biomasses with the aim of agronomic and environmental benefits
Source: PLoS One. 2017 May 11;12(5):e0176884. doi: 10.1371/journal.pone.0176884 (PMC5426627; doi:10.1371/journal.pone.0176884)
Supplement: S1 Table — 1The contents of P, K, Ca, Mg, Cu, Fe, Mn, and Zn were determined in extracts from the nitric-perchloric digestion procedure. 2Total content of B extracted with hot water. (DOCX) [file pone.0176884.s001.docx]

**Table S1.**

| Biomass | C | N | P^1^ | K | Ca | Mg | B^2^ | Cu | Fe | Mn | Zn |
| --- | --- | --- | --- | --- | --- | --- | --- | --- | --- | --- | --- |
|  | ------%------- | | ^__________^ g kg ^-1_____________^ | | | | ^_________________^ mg kg ^-1^ **^________________^** | | | | |
| Chicken manure | 25.2 | 4.9 | 25.9 | 19.3 | 117 | 4.6 | 38 | 61.8 | 3066 | 685 | 415 |
| Eucalyptus sawdust | 45.3 | 0.2 | 0.01 | 0.01 | 0.1 | 0.01 | 2.6 | 1 | 23.3 | 28.4 | 2.3 |
| Coffee husk | 43.3 | 2.9 | 1.1 | 22 | 1.9 | 0.2 | 17.9 | 16.2 | 222 | 47.7 | 5.5 |
| Sugarcane bagasse | 43.7 | 0.5 | 0.1 | 1.6 | 0.05 | 0.01 | 1.5 | 2.6 | 59.4 | 13.0 | 3.8 |
| Pine bark | 47.9 | 0.3 | 0.1 | 1.0 | 0.2 | 0.01 | 11.0 | 3.5 | 567 | 68.1 | 12.5 |

^1^The contents of P, K, Ca, Mg, Cu, Fe, Mn and Zn were determined in extracts, using the nitric-perchloric protocol as the digestion procedure. ^2^Total content of B extracted with hot water.
