# Supplementary figures and images for: Properties of biochar derived from wood and high-nutrient biomasses with the aim of agronomic and environmental benefits
Source: PLoS One. 2017 May 11;12(5):e0176884. doi: 10.1371/journal.pone.0176884 (PMC5426627; doi:10.1371/journal.pone.0176884)

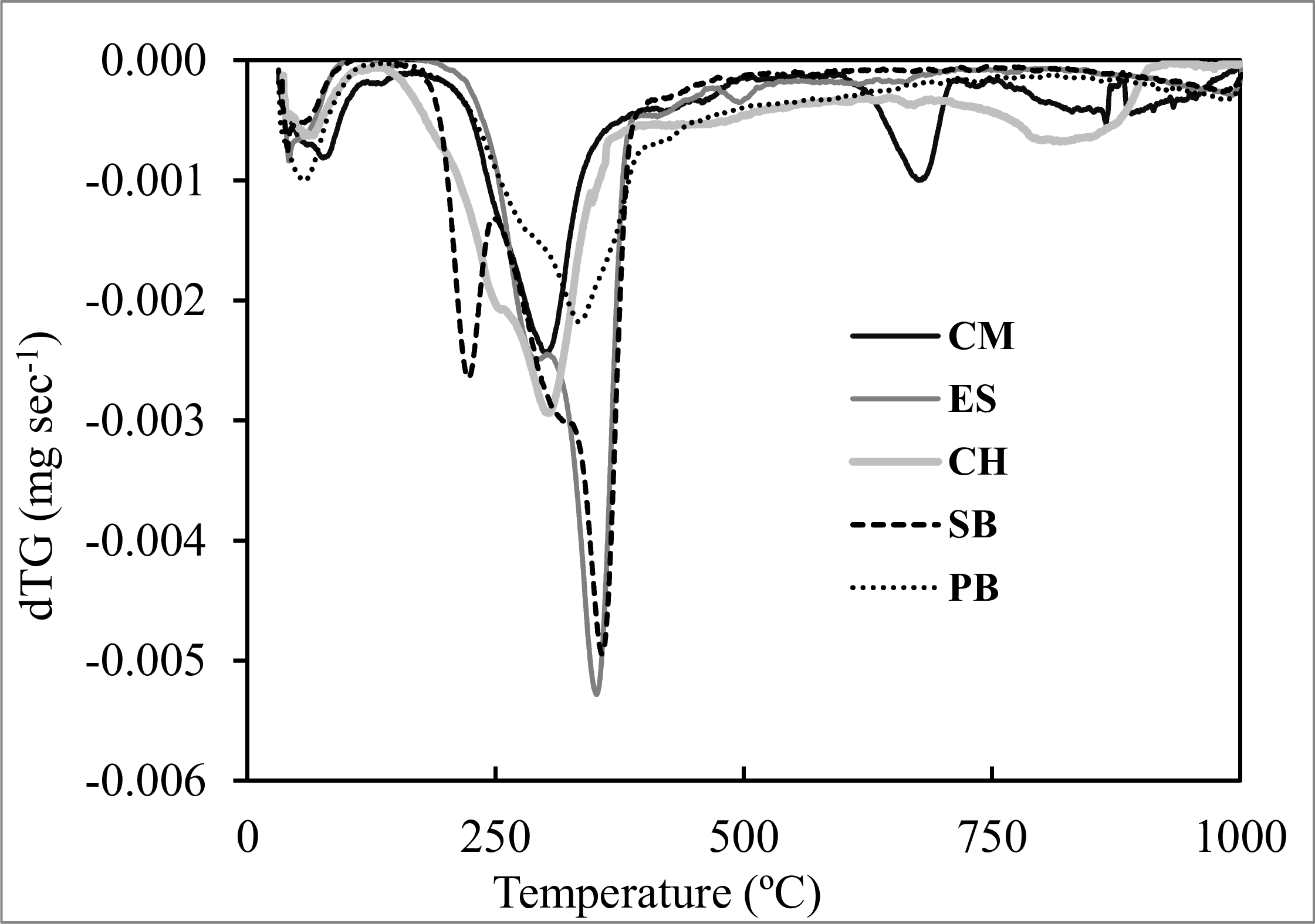

Supplement: S1 Fig — CM = chicken manure, SE = eucalyptus sawdust, CH = coffee husk, SB = sugarcane bagasse, and PB = pine bark. (TIF) [file pone.0176884.s002.tif]

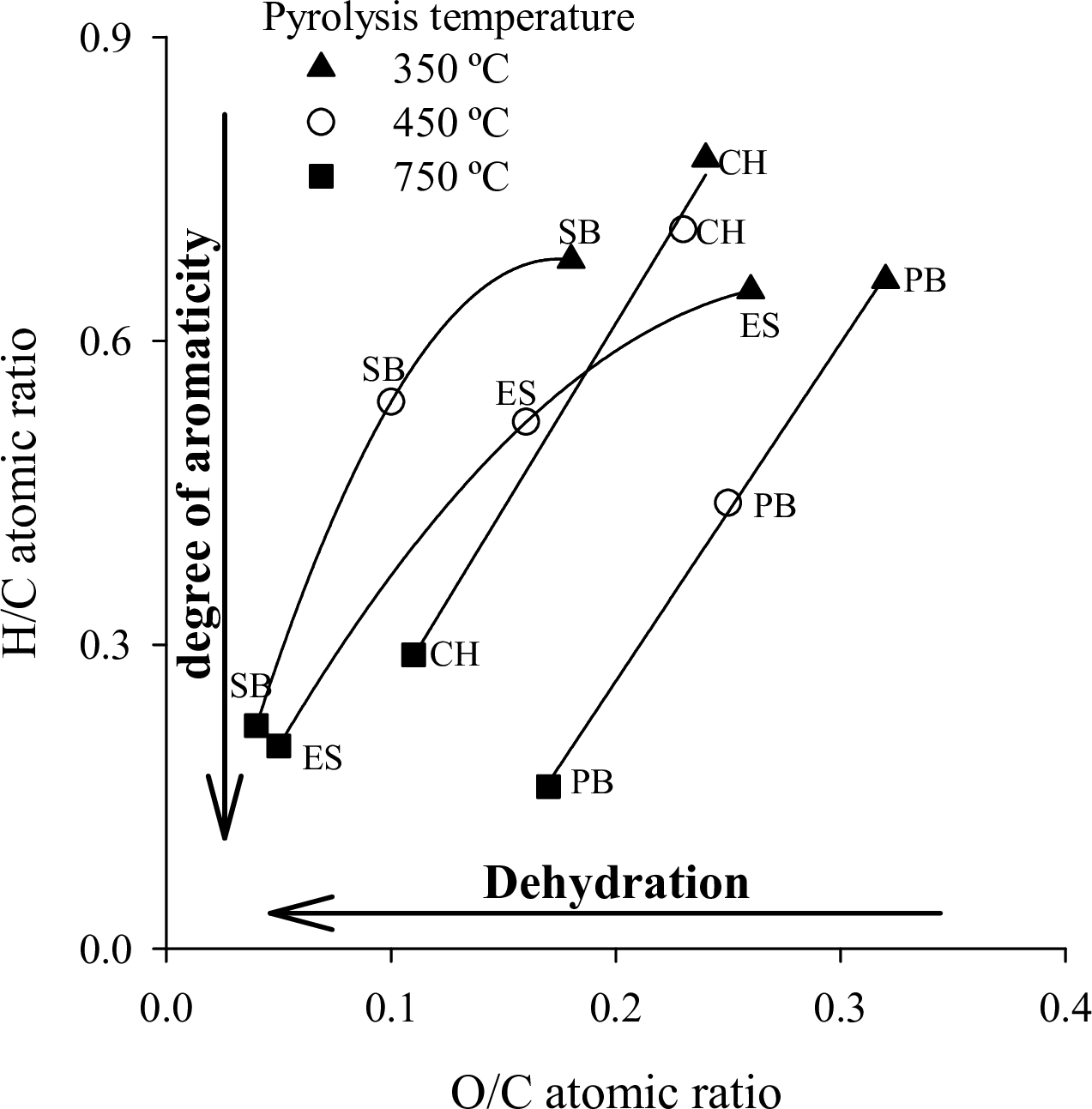

Supplement: S2 Fig — SE = eucalyptus sawdust, CH = coffee husk, SB = sugarcane bagasse, and PB = pine bark. (TIF) [file pone.0176884.s003.tif]
